# The 50s Cliff: Perceptuo-Motor Learning Rates across the Lifespan

**DOI:** 10.1371/journal.pone.0085758

**Published:** 2014-01-24

**Authors:** Rachel O. Coats, Andrew D. Wilson, Winona Snapp-Childs, Aaron J. Fath, Geoffrey P. Bingham

**Affiliations:** 1 School of Biomedical Sciences, Faculty of Biological Sciences, University of Leeds, Leeds, United Kingdom; 2 School of Social, Psychological and Communication Sciences, Leeds Metropolitan University, Leeds, United Kingdom; 3 Department of Psychological and Brain Sciences, Indiana University, Bloomington, Indiana, United States of America; University Medical Center Groningen UMCG, Netherlands

## Abstract

We recently found that older adults show reduced learning rates when learning a new pattern of coordinated rhythmic movement. The purpose of this study was to extend that finding by examining the performance of all ages across the lifespan from the 20 s through to the 80 s to determine how learning rates change with age. We tested whether adults could learn to produce a novel coordinated rhythmic movement (90° relative phase) in a visually guided unimanual task. We determined learning rates to quantify changes in learning with age and to determine at what ages the changes occur. We found, as before, that learning rates of participants in their 70 s and 80 s were half those of participants in their 20 s. We also found a gradual slow decline in learning rate with age until approximately age 50, when there was a sudden drop to a reduced learning rate for the 60 though 80 year olds. We discuss possible causes for the “50 s cliff” in perceptuo-motor learning rates and suggest that age related deficits in perception of complex motions may be the key to understanding this result.

## Introduction

The coordination of perception and action is intrinsic to numerous tasks of daily living such as walking, eating, dressing and driving a vehicle. Coordination is of primary concern to older adults because the loss of the ability to produce smooth coordinated muscle activity will lead to an increased risk of falling [1] and a decreased social and functional independence [2]. Perceptuo-motor learning, as well as performance, is essential for retaining independence, maintaining health or recovering from injury, and reducing the burden on caregivers and society at large. Older adults are also often required to re-learn coordination skills after injury, or learn new patterns of coordination such as fastening buttons with one hand or walking with walking sticks. Of course learning coordinative skills is relevant not only to older adults but adults of all ages. New tasks need to be learned on a continuous basis, especially with the advances in technology we are experiencing at the moment, and anyone can suffer from an injury that results in the need to re-learn previously attained coordinative skills. We recently discovered that older adults in their 70 s and 80 s learned a new pattern of coordination at half the rate of a group of 20 year olds [3]. Here we investigated the rest of the lifespan between these ages to determine how learning rates change with age.

**Figure 1:**
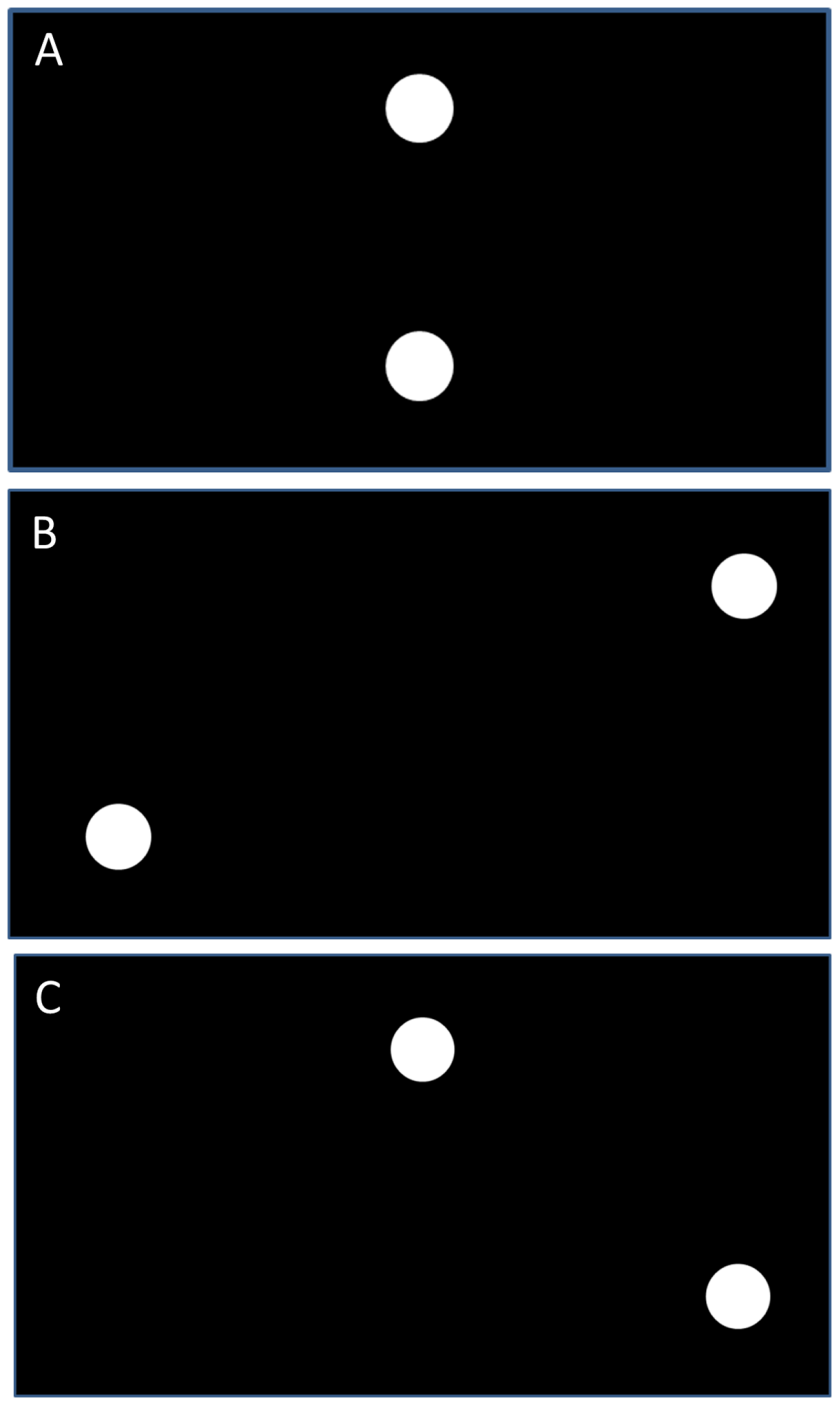
0°, 180° and 90° degrees. The oscillators at the three phase relations: 0° (a), 180° (b) and 90° (c).

Coordinated rhythmic movement (first described by Kelso, [4]) is a useful way of assessing perceptuo-motor learning. When two limbs, or two oscillators of any sort, move in phase (e.g. when two fingers move upwards and downwards at the same time) they are said to move at 0° relative phase. When they move in opposition (or anti-phase) they are at 180° relative phase. 0° is the most stable form of coordination. 180° can also be readily produced, but is less stable than 0° with people spontaneously switching to 0° at high frequencies. Both 0° and 180° can be produced with little intent or conscious effort [5]. They are also said to represent the intrinsic dynamics of the system and do not require learning [6]. Other coordination patterns such as 90° are difficult to produce in the absence of special circumstances (e.g. following metronomes) or training [5]–[8]. Wilson et al [11] showed that, without special training and perceptual learning, 90° relative phase is not perceived well and discriminated from different relative phases (other than 0° and 180°). Thus, it is not surprising that 90° relative phase is not easy to envisage. Imagine two oscillators/dots moving from left to right on a computer screen, one above the other. For 0° the dots move together – one always directly above the other and in the same direction (See Figure 1). For 180° the dots move in opposite directions. So when one moves left the other moves right and vice versa. In 90° (halfway between 0° and 180°) the dots are moving in the same direction only 50% of the time (see Figure S1). When the dots move from left to right one dot reaches the right side at the same time as the other one reaches the midpoint at peak speed. Many people see the pattern as one dot ‘chasing the other’ and always staying half the amplitude of the movement behind it. Due to the fact that it is hard to produce without training, we used the 90° relative phase pattern in the current experiment because we wanted to examine how people across the lifespan cope with learning a novel task; akin to the idea of a person with osteoarthritis learning to walk with a cane or a patient who has suffered from a bad fall or had a hip replacement re-learning to walk properly. It's not whether they can learn to produce 90 per se, but whether they can produce a new pattern or coordination.

Perceptual information is considered a crucial element of successful coordination [9]–[11]. In visual coordination studies where only vision is available to couple movements, and participants oscillate their limb to coordinate with the movement of another oscillator on a computer display [12]–[13], or another person [14]–[15], all the usual coordination dynamics are exhibited showing that coordination is mediated by perception. From this perception-action perspective on coordination it follows that rhythmic production of 90° is difficult because participants are unable to perceive and recognise it. This is not to say that observers cannot learn to perceive and produce it with training, they can [11], [16], but stable production requires stable access to perceptual information and this needs to be learned. Additional evidence for the central role of perception comes from studies using lissajous displays (position-position plots), where transformed visual feedback information leads to improved performance of 90° relative phase [17]–[18] and studies where 90° performance is possible when the perceived phase relation displayed is transformed to look like 0° [13], [19].

Most research into perceptuo-motor performance and learning has been carried out on young adults. Less is known about older adults, and even less about those between these two age groups. Serrien et al. [7] examined bimanual performance of 0° and 180° relative phase in young (mean age 24) and older (mean age 75) adults and found performance deteriorated for the older adults when dissimilar limbs were used, and this was enhanced in the anti-phase mode. Swinnen et al [6] investigated the performance of bimanual 90° rhythmic movements with lissajous feedback by nine older (mean age 73) and nine younger (mean age 19) participants. They found that older adults showed lower performance levels than the younger adults across acquisition and retention and were more variable. In respect to learning, the younger adults showed a steep decline in error on day 1, but this decrease was equivalent to that of the older adults on day 2. Wishart et al. [20] also examined bimanual performance and learning of 90° by older and young adults with lissajous feedback. Both groups could learn to produce 90°, although the older adults were not as consistent as the younger adults, and only benefitted from concurrent visual feedback at the end of day 3, whereas the younger adults benefitted on day 1.

In a recent study, Coats et al [3] sought to fill a gap in the existing literature by quantifying learning rates and changes in learning rates. They examined unimanual/visual coordination without transformed feedback in older and younger adults. They found that older adults exhibited learning rates that were half those of their younger counterparts and suggested that, while there are likely to be a number of factors underlying these age differences, emerging deficits in motion perception may be key. In the current study, we set out to determine how and when this change in learning rate occurs by examining the rest of the lifespan. Is there a gradual change in performance with age? Or is there a sudden drop, and if so at what age? Participants followed the exact same procedure as those in Coats et al [3]. 9–10 participants from each age decade from 20 s to 80 s were tested on a visual coordination task with concurrent feedback on performance. Participants were tested on their baseline ability to move at 0°, 90°, and 180°, trained at 90° over 5 sessions, and re-evaluated at post test and retention. The data yielded learning curves that were fit by a model and used to estimate learning rates, separately for each of the age groups. The majority of the data from the 20, 70 and 80 year olds have already been published [3] so we would expect the same learning rates in these groups (with rates of the older groups being half that of the younger group), but how learning rates change across the lifespan (30 s, 40 s, 50 s, 60 s in addition to 20 s, 70 s, and 80 s) remains to be seen.

## Method

### Ethics Statement

This study was approved by the Institutional Review Board at IU Bloomington. Written informed consent was obtained from all participants.

### Participants

Ten young adult participants in their 20 s (3 male; mean age 22), 10 participants in their 30 s (5 male; mean age 33), 10 in their 40 s (3 male; mean age 44), 9 in their 50 s (2 male; mean age 54), 10 in their 60 s (8 male; mean age 67), 10 in their 70 s (3 male; mean age 74) and 9 in their 80 s (3 male; mean age 84) participated in the study. The majority of participants were recruited from the Indiana University or wider Bloomington community including the YMCA, IU Tennis Center, Meadowood retirement community (six 70 year olds and seven 80 year olds), and from fliers placed around the IU campus. Four of the 60 year olds and one 83 year old were recruited from Sandbach Rugby Club, UK. All participants had normal or corrected-to-normal vision. All participants were naïve to the experimental questions and their 90° relative phase production was worse than their 0° and 180° relative phase production prior to training. Measures of cognitive function were collected from the older adults (70 s and 80 s) using the Short Portable Mental Status Questionnaire [21] and all participants scored within the range of normal mental functioning. Data from all the 20 year olds, nine 70 year olds and eight 80 year olds have already been published [3].

### Procedure

The procedure was identical to that used in Coats et al. [3]. Participants sat in front of a Dell Latitude 15′′ laptop, with the monitor set to a resolution of 1024×768 and a refresh rate of 60 Hz that was connected to a Logitech Force 3D Pro joystick (force feedback feature disabled) via USB. The computer presented a display showing two white dots, one above the other, on a black background. In the display, the dots oscillated horizontally from side-to-side. Except for task demonstrations, the top dot was under the control of the computer, while the bottom dot was under the control of the participant via the joystick. All participants used their preferred hand to control the joystick. The amplitude of movement of each dot was 300 pixels and each dot was 60 pixels in diameter. Stimulus presentation, data recording and all data analysis was handled by a custom Matlab toolbox written by ADW, incorporating the Psychtoolbox (http://psychtoolbox.org) [22]–[24].

There were four Assessment sessions (Baseline x2, Post Training and Retention) and five Training sessions. These sessions were spread over eight separate days (not necessarily consecutive but within a nine week period). We conducted two baseline sessions primarily to ensure that task novelty was not too large a contributing factor to baseline performance. The measures that we report for “baseline” represent the averaged performance during these two sessions. In the assessment sessions, participants first viewed an 8 s demonstration of the 0° target relative phase then they attempted to produce 0° five times by moving the joystick from side to side. All movement trials were 20 s in duration. The first trial was practice and online feedback was given. The feedback was a “hot/cold” signal where the dot that the participant controlled turned green within a certain range and is described below and in Wilson et al. [25]. Participants were told prior to the practice trial that when the feedback was “on” during the practice trial then they were moving successfully. During the subsequent trials, no feedback was given (participants were apprised that no feedback would be available during these trials). This procedure was then repeated for the 180° and 90° target relative phase conditions.

In each of five training sessions, participants performed ten 20 s 90° trials, with an 8 s demonstration before every trial. So, participants performed a total of 50 trials over five separate days. During each trial, online feedback was provided by changing the colour of the person-controlled dot from white to green when the participant was moving at 90°, +/− an error bandwidth. The error bandwidth was faded (decreased) across sessions when performance reached a certain threshold. The level participants were started on in the first training session was dependent on performance at baseline: data were analysed to see at which error bandwidth (from +/−35° to +/−10° in 2.5° intervals) the participant could perform the task (i.e. stay within this bandwidth) 50% of the time, and this was the level at which they started. After subsequent training sessions, data were again analysed in a similar way, and the error bandwidth was altered for the next training session (but only by a maximum of 5° each time) if performance improved. Participants were informed of this change. If performance did not improve the error bandwidth remained the same. Changes to the error bandwidth, which drives learning, were therefore self paced.

### Data Analysis

The two position time series from each trial were filtered using a low-pass Butterworth filter with a cut-off frequency of 10 Hz and numerically differentiated to yield a velocity time series. These were used to compute a time series of relative phase, the key measure of coordination between the two dots.

To assess the stability of the coordination over the course of a trial, we used a measure of proportion of time on task (PTT). The measure is the proportion of time during a trial that the relative phase falls within a +/−20° window of the target relative phase (e.g. 90°). We averaged PTT, for each participant, over the trials performed in a given condition. We chose PTT as the primary measure because, in human movement, stability is not independent of mean relative phase; so measures that simply assess overall movement variability (e.g. the standard deviation of mean relative phase or mean vector length) are confounded with the actual relative phase produced. Coordination stability at 90° can be artificially elevated if participants spend time at other locations (e.g. 0° or 180°), which they do as these locations are natural attractors [5], (see Wilson et al. [13] for an extended analysis of this problem). Proportion of time on task allows us to address this problem (see Snapp-Childs, et al. [10] for an explicit comparison of the two methods). It is simply the proportion of the relative phase time series that falls within the range of the target phase +/− a tolerance (e.g. of 20°), thus summarizing the data of interest (consistency and accuracy) and eliminating the confound. This measure ranges from 0–1 and validly measures stability of coordination at the required relative phase in a single number [11], [25].

We examined the differences between performance pre and post-training at 0°, 90° and 180° for all age groups using mixed design ANOVAs with session (baseline, post-test and retention) and relative phase (0°, 180° and 90°) as repeated measures factors, and group (20-year olds, 30-year olds, 40 year-olds, 50 year-olds, 60 year-olds, 70-year olds, 80-year olds) as a between-subjects factor. Further mixed ANOVAs (group × session) were used to look at relative phase separately, and paired t-test/one-way ANOVAs utilised to examine interactions.

We also examined learning rate. Exponential functions were fitted to the data. The functions were of the form:
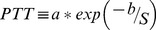
(1)where PTT is ‘Proportion of Time on Task’, S is session (1 =  baseline and 7 =  post-training), and a and b are parameters. The function was fitted in three different ways and results were compared to be sure they were essentially the same. First, the function was fit to the means separately for each age group using Quasi-Newton estimation in Systat 5.2. Secondly, the PTT means and session numbers were transformed as follows:



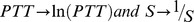



Least squares linear regression was used to fit a line to the relation between the two sets of transformed values, again separately for each group. Third and finally, transformation and linear regression was used again applied to the combined individual participant data for each group, However, in this last case, we also used multiple linear regression to test differences in slope and intercept between the groups taken two at a time. Once values for a and b parameters were identified (and judged to be equivalent) we computed the first derivative of the function in Equation (1) and evaluated it at Session 1 to get a value for learning rate.

## Results

**Figure 2:**
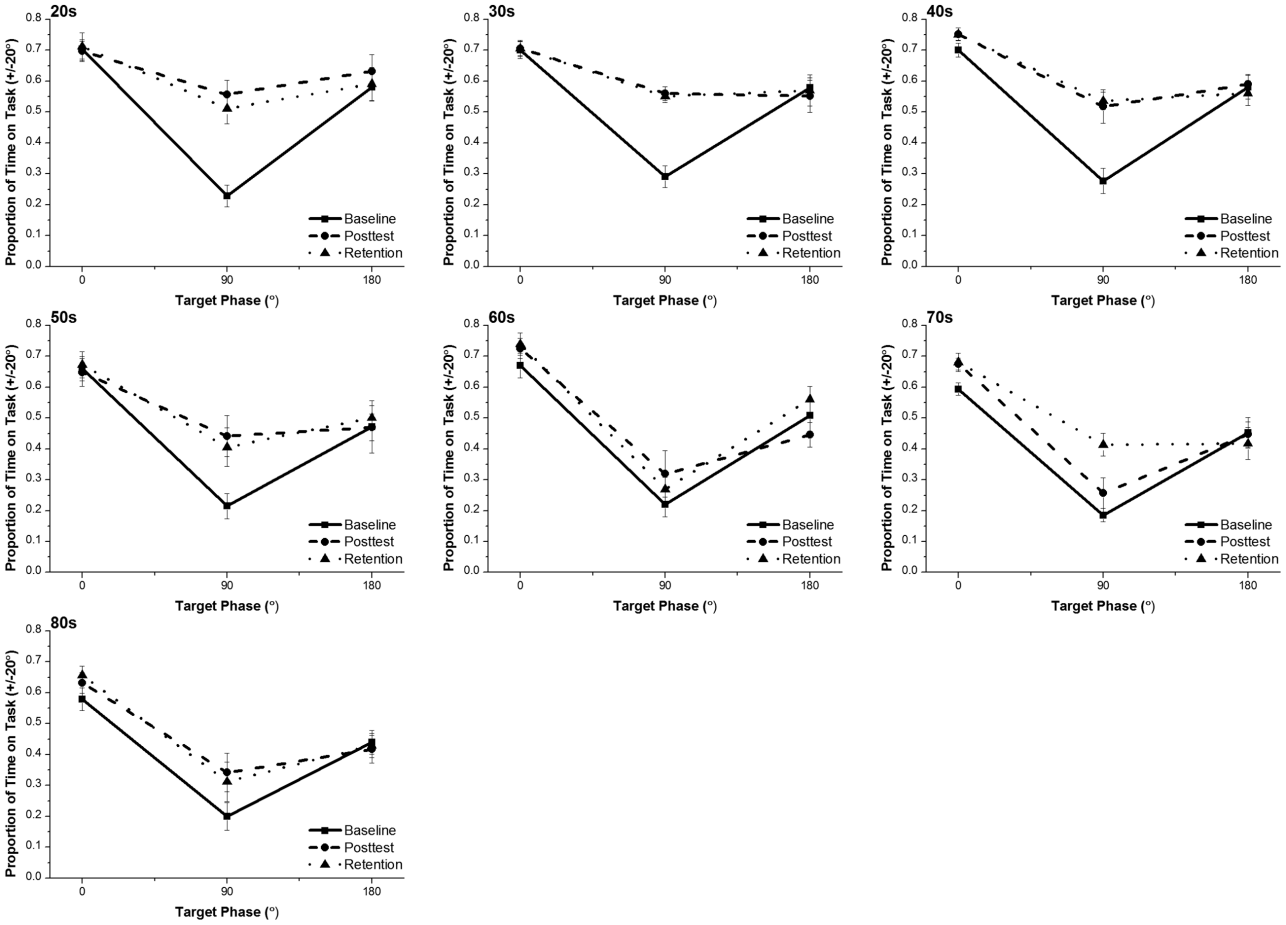
Proportion of time on task across age, relative phase, and assessment session. Proportion of time spent within 20° of the target mean relative phase (0°, 90°, or 180°) across the baseline *(solid line),* post-training *(dash-dot line)* and retention *(dotted line)* sessions for all age groups.

Figure 2 shows the performance of all groups at baseline, post-test and retention for all conditions, measured as proportion of time-on-task (PTT) using a +/−20° window. The figure shows that all groups performed equally poorly at baseline for the 90° pattern, and any group differences at 0° and 180° at baseline appear small if present at all. All groups were better at performing 0° than 180°in all three sessions. For the 90° pattern, it is evident that the younger groups (20 s, 30 s, 40 s and 50 s) showed a greater improvement between baseline and post-test than the older groups (60 s, 70 s and 80 s).

We performed 3-way mixed design ANOVA with session (baseline, post-test and retention) and relative phase (0°, 180° and 90°) as repeated measures factors, and group (20-year olds, 30-year olds, 40 year-olds, 50 year-olds, 60 year-olds, 70-year olds, 80-year olds) as a between-subjects factor. Group was significant [F(6, 61)  = 5.25 ; p<0.01, η_p_^2^ =  0.34], with the 20, 30 and 40 year olds (mean time-on-task  = 0.58, 0.58 and 0.59 respectively) performing better than the 50 and 60 year olds (mean time-on-task  = 0.50 for both), who were better than the 70 and 80 year olds (mean time-on-task  = 0.46 and 0.45). A significant main effect of session was also identified [F(2, 122)  = 55.37 ; p<0.001, η_p_^2^ =  0.48], with performance at post-training (mean time-on-task  = 0.54) and retention (mean time-on-task  = 0.55) being better than performance at baseline (mean time-on-task  = 0.47). Relative phase was significant [F(2,122)  = 217.9 ; p<0.001, η_p_^2^ = 0.78] with performance at 0° (mean time-on-task  = 0.69) being better than performance at 180° (mean time-on-task  = 0.52) and 90° (mean time-on-task  = 0.36). Significant interactions were found between session and relative phase [F(4, 244)  = 36.24 ; p<0.001, η_p_^2^ =  0.37], but not between session and group or relative phase and group. The 3-way interaction between group, session and relative phase was also significant [F(24, 244)  = 2.73; p<0.01, η_p_^2^ =  0.21] so further analyses were required to reveal the nature of this interaction.

### 90°

First, we wanted to determine whether all groups could learn the 90° relative phase pattern. Although all groups seem to improve between baseline and post-test, it is clear that the younger participants (20s–50s) show a larger increase in time-on-task between baseline and post-test than the older participants (60s–80s). A repeated measures ANOVA on the 90° performance data at baseline, post-test and retention revealed a significant main effect of group [F(1, 61)  = 5.80 ; p<0.01, η_p_^2^ = 0.36] and a significant main effect of session [F(2,122)  = 67.30 ; p<0.001, η_p_^2^ = 0.53]. There was also a significant group by session interaction [F(2,122)  = 2.79; p<0.01, η_p_^2^ = 0.22].

To further analyze the main effects and interactions, we performed paired t-tests which showed that there were significant improvements in performance at post-training compared to baseline for the 20 s [t(9)  = −7.34 ; p<0.001], 30 s [t(9)  = −8.79; p<0.001], 40 s [t(9)  = −4.77; p<0.01], 50 s [t(8)  = −2.80; p<0.05] and 80 year olds [t(8)  = −3.75 ; p<0.01] but not for the 60 s [t(9)  = −1.93; p = 0.09] or 70 year olds [t(9)  = −1.44 ; p = 0.18]. However, examination of baseline versus retention [t(9)  = −4.40 ; p<0.01] revealed significant improvement from baseline for the seventy year olds, but not for the 60 year olds [t(9)  = −0.87; p = 0.41]. No differences were found between post-training and retention in separate tests for each age group apart from the 70 year olds [t(9)  = −2.47; p<0.05].

Separate one-way ANOVAS and pairwise comparisons (with Bonferroni corrections applied) on the baseline and post-training data revealed no significant differences between any of the groups at baseline, but a significant difference between the 70 year olds and 20 year olds [p<0.01], 30 year olds and [p<0.01] 40 year olds [p<0.05] at post-training (in all cases the younger group performing better).

### Learning Rates for 90° Relative Phase

**Figure 3:**
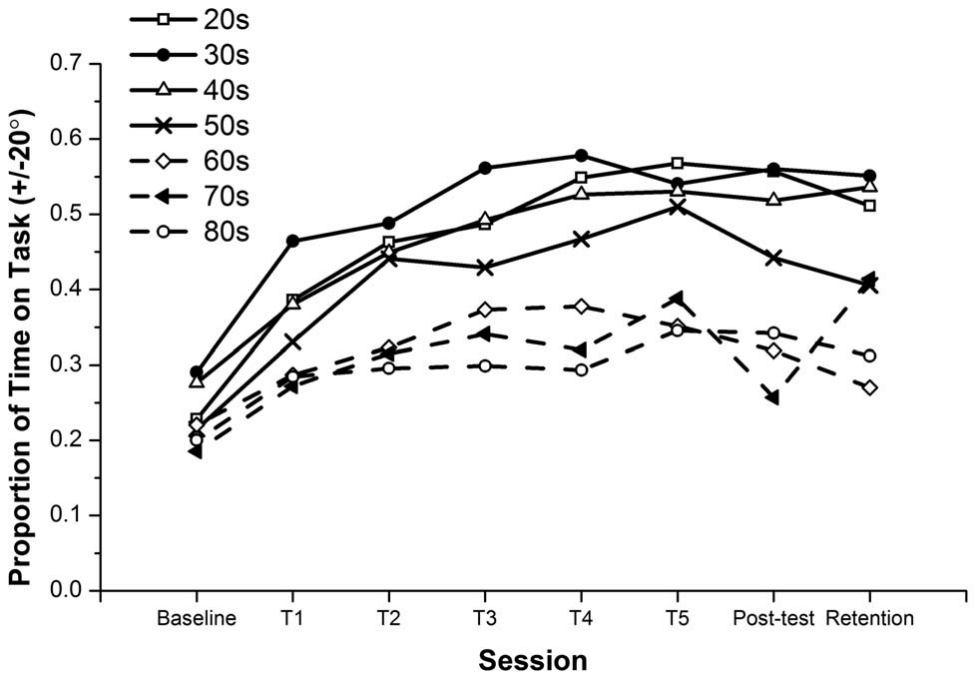
Learning 90°. Proportion of time on task for each age group across all training and assessment sessions.

As well as examining potential differences in the amount of learning between baseline and post-training or retention, we also wanted to determine whether the learning *rates* between the groups were different and if so, exactly how different. Figure 3 shows the mean learning curve for each of the age groups performing 90° relative phase across all sessions (apart from retention). Exponential functions were fitted to the data. The functions were of the form:
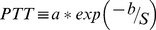
(2)


where PTT is ‘Proportion of Time on Task’, S is session (1 = baseline and 7 =  post-training), and a and b are parameters. As mentioned in the methods section, the function was fitted in three different ways and results were compared to be sure they were essentially the same. First, the function was fit to the means separately for each age group using Quasi-Newton estimation in Systat 5.2. This yielded r^2^>0.80 in all cases. The values for parameter a were 0.664 for the 20 s and 30 s, 0.597 for the 40 s, 0.554 for the 50 s, 0.391 for the 60 s, 0.369 for the 70 s and 0.360 for the 80 s. For parameter b they were 1.073, 0.750, 0.802. 0.919, 0.556, 0.607 and 0.577 for each group respectively from 20 s through 80 s. Secondly, the PTT means and session numbers were transformed as follows:



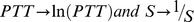



Least squares linear regression was used to fit a line to the relation between the two sets of transformed values, again separately for each group. The r^2^ were 0.98, 0.96, 0.98, 0.90, 0.56, 0.69, and 0.89 for each group respectively from 20 s through 80 s. All were significant p<0.05 or better.

Third and finally, transformation and linear regression was used again applied to the combined individual participant data for each group. However, in this last case, we also used multiple linear regression to test differences in slope and intercept between the groups taken two at a time [26]. Both slope and intercept differences yield a difference in learning rate as both form part of the relevant equation, so differences in either are summarised below. The results of the comparisons of 20 s and all other groups were significant (p<0.01) with differences in slope (20 s vs. all groups apart from the 50 s) and intercept (20 vs. all groups apart from the 30 s). The 30 s were significantly different from the 50 s, 60 s, 70 s and 80 s in terms of intercept (p<0.01), as were the 40 s and 50 s compared to the 60 s, 70 s and 80 s. The three older groups were not significantly different from each other in terms of either slope or intercept.

In the two sets of linear regression analyses (i.e. using means and individual participant data), the resulting linear equations were transformed back into the form of Equation (1). The values found for the parameters a and b using all three approaches were essentially the same. Then, in each case, we computed the first derivative of the function in Equation (1), that is:
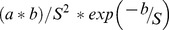
(3)


**Figure 4:**
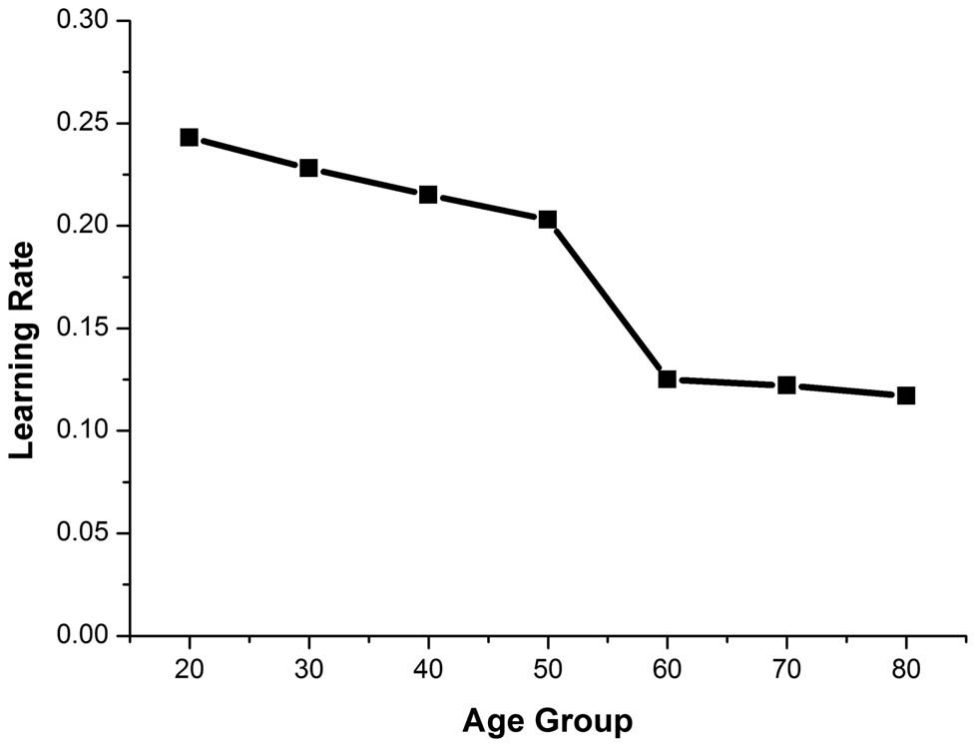
Learning rates for all age groups. Note the dramatic drop from 50

We evaluated this derivative at S = 1 to derive an estimate of the learning rate. Again, the resulting estimates were nearly identical using all three fitting methods. The resulting learning rates were: 20 s = 0.243, 30 s = 0.228, 40 s = 0.215, 50 s = 0.203, 60 s = 0.125, 70 s = 0.122, 80 s = 0.117 (reporting the mean of the results of the 3 methods in each case). You can see from Figure 4 that there is a steep drop in learning rate between the 50 s and 60 s. The learning rates for the 3 older groups were almost exactly half that for young adults in their 20′s.

### 0° and 180° Relative Phase

We wanted to determine if there were any changes in performance for the untrained coordination patterns, 0° and 180°, as a function of age group and/or training at 90°. Learning 90° does not typically transfer to either 0° or 180°, because learning 90° entails learning a different perceptual coupling [16].

A repeated measures ANOVA on 0° performance revealed no significant main effect of group (p>0.05), but there was a significant main effect of session with performance being higher at post-training (mean time-on-task  = 0.69) than baseline (mean time-on-task  = 0.66) [F(1,61)  = 10.78 ; p<0.01, η_p_^2^ =  0.15]. There was also an interaction between group and session [F(6,61)  = 2.41 ; p<0.037, η_p_^2^ =  0.19]. To further analyze the main effects and interactions, we performed paired t-tests which showed that there were significant improvements in performance at post-training compared to baseline for the 60 [t(9)  = −2.72; p<0.05] and 70 year olds [t(9)  = −3.01 ; p<0.05], but not any of the other groups (p>0.05). Separate one-way ANOVAS and pairwise comparisons (with Bonferroni corrections applied) on the baseline and post-training data revealed no significant differences between any of the groups at either baseline or post-test (p>0.05).

A repeated measures ANOVA on 180° performance revealed a significant main effect of group [F(1,61)  = 3.07 ; p<0.05, η_p_^2^ =  0.23] with the mean time-on-task of each group as follows: 20 s = 0.61, 30 s = 0.57, 40 s = 0.59, 50 s = 0.47, 60 s = 0.48, 70 s = 0.45 and 80 s = 0.43. There was no main effect of session or interaction (both p>0.05).

Overall therefore, all age groups were equally able to perform 0° coordination, showing that poorer performance by the older adults in the 90° condition was not a function of problems using the joystick or seeing the display. However, the older adult groups (50 s, 60 s, 70 s and 80 s) performed 180° less well than the younger participants (20 s, 30 s, and 40 s). 0° is an easy coordination to maintain because the relative phase is clearly perceived [13], [27]. The two oscillators move together always in the same direction with no speed difference between them, so that the ability to resolve the phase relation is good throughout the motion [9], [28]. On the other hand, 180° yields oppositely directed motion throughout, and the relative speeds of motion vary from zero to a peak at the mid-point of motion. This variation makes relative phase harder to perceive than at 0°. This pattern in the data shows that people over 50 performed worse when the coordination requirements increased in complexity. This suggests that the difference in learning rates at 90° may be related to the visual perception of coordination, i.e. relative phase.

## Discussion

In a previous study [3] we found that older adults show reduced learning rates when learning a new pattern of coordinated rhythmic movement. The purpose of this study was to extend that finding by examining the performance of all ages across the lifespan from 20 s through to 80 s to determine how learning rates change with age. We tested whether adults could learn to produce a novel coordinated rhythmic movement (90° relative phase) in a visually guided unimanual task. We also determined learning rates to quantify changes in learning with age and to determine at what ages the changes occur. We found, as before, that learning rates of participants in their 70 s and 80 s were half those of participants in their 20 s. We also found that learning rates for participants in their 30 s were significantly greater than rates for participants aged 50 and above. Participants in their 40 s and 50 s were faster learners than participants in their 60 s, 70 s and 80 s, and the three oldest groups did not differ from one another in terms of how fast they learned. What was most interesting was the gradual slow decline in learning rate with age until approximately age 50, when there was a sudden drop to a reduced learning rate for the 60 though 80 year olds. The reduced learning rate of the older participants was about half the learning rate of the younger participants.

The mechanisms underlying the reduced learning rates in the 60, 70 and 80 year olds are likely to be multiple, but as we mentioned in our previous paper [3] we think deficits in motion perception are key. If you cannot perceive 90° how can you be expected to produce it? In addition to examining learning at 90°, we also tested performance of 0° and 180° before and after training at 90°. We found that the groups did not differ in their ability to perform 0°, but that the 50, 60, 70 and 80 year olds showed reduced performance at 180° compared to participants in their 20 s, 30 s and 40 s. According to the Bingham model of rhythmic coordination [9], [10], [28] the difference in stability of performance between 0° and 180° is produced by differences in the relative speeds of the two oscillators (empirically confirmed by Snapp-Childs, et al. [10]). For 0°, the dots move together and the relative speed difference between them is zero. In 180°, the relative speed difference varies over the cycle to be zero at the end points and greatest when the dots pass each other in the middle travelling in opposite directions. Speed differences like these condition the ability to see the relative directions of motion that, according to the model, specify the relative phase. Learning to produce 90° entails perceptual learning, namely, learning to perceive 90°. This has been shown by Wilson et al. [11] who found that participants were able to perform 90° once they had learned to see it, without any actual motor practice of the coordinative movements. 90° is specified by a more complex pattern of speeds and positions, rather than merely relative direction. This more complex spatial-temporal pattern is apparently difficult for older participants to learn to perceive. Further research might investigate whether training older participants to perceive 90° could compensate for the 50 s cliff we found in learning how to produce it.

Aging has also been shown to negatively affect a variety of tasks requiring visual perception of motion (see Andersen [29] for a review). For example, visually discriminating speeds is difficult for older adults [30]–[31] and aging detrimentally affects the ability of older adults to visually perceive the 3-D shape of objects defined by motion [32]–[33]. Changes such as these are likely to be underpinned by general changes in cortical function that occur with age, such as decreases in neuronal inhibition leading to reduced centre-surround antagonism in visual cortex and less finely resolved motion detection systems [34]–[36].

Why we found the sudden drop in learning rate at around age 60 remains unclear. Perhaps we go through specific neurological changes at this age, or the behavioural effects of cortical changes are non-linear and at 60 become suddenly more apparent. Interestingly, the fact that learning rates showed a sudden drop at 60, with the 50 year olds remaining at the top of the metaphorical cliff, was not coincident with the decrement that we found in performance of the 180° relative phase task, where the 50 year olds showed a reduced performance level relative to younger participants and looked very similar to those in their 60 s, 70 s and 80 s. Perhaps the ability to perform 180° degrees acts as a marker for subsequent deterioration in learning rate at 90°, as the ability to deal with more complex motion patterns begins to decline.

In conclusion, we have identified a dramatic change in perceptuo-motor learning rates of a standard laboratory task (coordinated rhythmic movement) at around the late 50 s or early 60 s. This is surprisingly early, and has implications for movement rehabilitation following stroke (which disproportionately affects older adults). Research on older adults typically focuses on older age groups (e.g. 60 s and up); these data suggest that it is vital to take a lifespan view in order to identify earlier, functionally relevant changes.

## Supporting Information

Figure S1 **90°.** Shows 90° phase relation.
(GIF)
