# Supplementary figures and images for: The 50s Cliff: Perceptuo-Motor Learning Rates across the Lifespan
Source: PLoS One. 2014 Jan 24;9(1):e85758. doi: 10.1371/journal.pone.0085758 (PMC3901653; doi:10.1371/journal.pone.0085758)

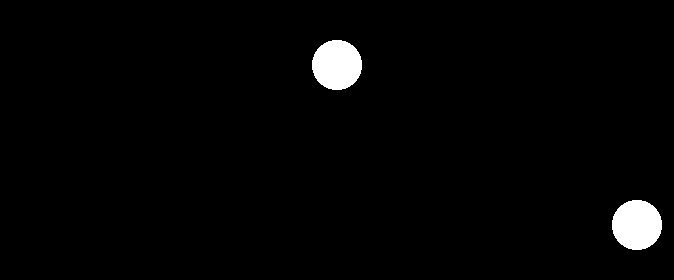

Supplement: Figure S1 — 90°. Shows 90° phase relation. (GIF) [file pone.0085758.s001.gif]
